# Critical Role of Estrogens on Bone Homeostasis in Both Male and Female: From Physiology to Medical Implications

**DOI:** 10.3390/ijms22041568

**Published:** 2021-02-04

**Authors:** Emmanuelle Noirrit-Esclassan, Marie-Cécile Valera, Florence Tremollieres, Jean-Francois Arnal, Françoise Lenfant, Coralie Fontaine, Alexia Vinel

**Affiliations:** 1 I2MC, INSERM UMR 1297, University of Toulouse III, F-31000 Toulouse, France; noirrit.e@odonto-tlse.eu (E.N.-E.); marie.valera@inserm.fr (M.-C.V.); tremollieres.f@chu-toulouse.fr (F.T.); jean-francois.arnal@inserm.fr (J.-F.A.); francoise.lenfant@inserm.fr (F.L.); Coralie.fontaine@inserm.fr (C.F.); 2 Department of Pediatric Dentistry, Faculty of Dental Surgery, University of Toulouse III, F-31000 Toulouse, France; 3 Menopause and Metabolic Bone Disease Center, Hôpital Paule de Viguier, University Hospital of Toulouse, F-31000 Toulouse, France; 4 Department of Periodontology, Faculty of Dental Surgery, University of Toulouse III, F-31000 Toulouse, France

**Keywords:** bone remodeling, sexual dimorphism, estrogens, estrogen receptor, nuclear effects, ERαMISS

## Abstract

Bone is a multi-skilled tissue, protecting major organs, regulating calcium phosphate balance and producing hormones. Its development during childhood determines height and stature as well as resistance against fracture in advanced age. Estrogens are key regulators of bone turnover in both females and males. These hormones play a major role in longitudinal and width growth throughout puberty as well as in the regulation of bone turnover. In women, estrogen deficiency is one of the major causes of postmenopausal osteoporosis. In this review, we will summarize the main clinical and experimental studies reporting the effects of estrogens not only in females but also in males, during different life stages. Effects of estrogens on bone involve either Estrogen Receptor (ER)α or ERβ depending on the type of bone (femur, vertebrae, tibia, mandible), the compartment (trabecular or cortical), cell types involved (osteoclasts, osteoblasts and osteocytes) and sex. Finally, we will discuss new ongoing strategies to increase the benefit/risk ratio of the hormonal treatment of menopause.

## 1. Introduction

Far from inert, bone is a highly dynamic tissue undergoing constant remodeling regulated by numerous parameters and, among others, estrogens are of major importance. Estrogens are steroidal compounds derived from cholesterol. There are four identified estrogens: 17β-estradiol (E2), the most powerful and well known, estrone (E1) produced during menopause, estriol (E3) released during pregnancy by the placenta and estetrol (E4) synthesized by fetal liver [1]. In women, E2 is synthetized in the ovaries from puberty to menopause. E2 is responsible for the development of primary and secondary sexual characteristics in women but it is also produced in men via aromatization of testosterone in the testes (20%) and peripheral tissues (80%) [2]. It is now admitted that estrogens are key regulators of bone remodeling in both sexes [3]. Although its multifactorial origin is not quite fully elucidated (direct and indirect effects of pubertal sexual steroids, role of autosomal genes), sexual dimorphism of the skeleton mass and architecture is well known: men have wider long bones than women and their vertebrae have a higher bone volume and thus a higher trabecular bone mineral density (BMD). The review aims to describe bone turnover regulation by estrogens and their receptors and the main differences and their common characteristics in both females and males.

### 1.1. Bone Physiology

Bone is a metabolically active and complex tissue conferring hardness and resistance to the skeleton. It exhibits four major roles: mechanical, since it supports the organism and allows locomotion, protection of vital organs (brain, heart, lungs, etc.) and hematopoietic marrow, metabolic, as the reservoir of calcium, phosphorus and mineral ions and endocrine via the production of osteocalcin and FGF23 [4].

Every bone in the skeleton is made of two types of tissue, cortical bone and trabecular bone. Cortical bone constitutes the compact external wall of every bone as well as the diaphysis of long bones; it represents 80% of the skeleton. The remaining 20% is made of trabecular bone, composing the end of long bones and flat bones, and is made of a tridimensional network of interconnected trabeculae surrounded by bone marrow. Bone is a highly specialized connective tissue comprising a mineralized organic substance with an extracellular matrix with collagen fibers and non-collagenic proteins, a calcium hydroxyapatite crystal mineral fraction and bone cells [5].

### 1.2. Bone Cells

Bone contains two main types of highly differentiated cells, the osteoclasts responsible for bone resorption and osteoblasts that are in charge of bone production.

Resulting from the fusion of hematopoietic mononuclear progenitors, osteoclasts are giant multinucleated cells that represent less than 1% of bone cells. Two molecules are necessary to induce osteoclastogenesis, macrophage colony-stimulating factor (M-CSF) and receptor activator of nuclear factor kappa-B ligand (RANKL) [6]. M-CSF produced by bone marrow stromal cells is essential for the survival and proliferation of osteoclast precursor monocytes/macrophages. RANKL is a resident membrane protein at the surface of osteoblasts, specifically recognized by its receptor RANK present at the surface of bone marrow monocytes/macrophages [6]. Osteoclasts are the only cells able to resorb bone tissue. Activated osteoclasts form a hermetic compartment between themselves and the bone surface in which they release protons and chloride (Cl^−^) responsible for bone demineralization. Then, the exposed organic phase is degraded by cathepsin-K and matrix metalloproteinases that are also secreted by osteoclasts [7].

Osteoblasts are bone-forming cells, accounting for 4 to 6% of bone-residing cells. They derive from mesenchymal stem cells following the expression of several transcription factors, including RUNX2 and OSX [8]. Mature osteoblasts secrete bone matrix proteins like osteocalcin (OCN), bone sialoproteins I and II and type 1 collagen [9]. During bone formation, osteoblasts are responsible for the secretion of new organic bone matrix and its mineralization [9,10]. In addition, they have a paracrine effect on osteoclasts by expressing RANKL and producing M-CSF [10,11].

Osteocytes are the last stage of osteoblastic differentiation; they represent 95% of bone cells. They are stellate cells with numerous dendritic processes embedded in the mineralized bone matrix. During the transition from mature osteoblasts to osteocytes, the expression of OCN, bone sialoproteins and type I collagen is strongly repressed and new markers such as dentin matrix protein-1 (DMP1) and sclerostin are highly expressed [12]. Osteocytes are mechanosensory cells able to sense and respond to mechanical forces. They are tightly connected to one another via their dendritic processes, which constitute a tridimensional network within the mineralized bone matrix [13].

### 1.3. Bone Modeling and Remodeling

Bone modeling determines the development and maintenance of bone shape during skeletal growth. It starts from the very beginning of fetal bone formation and lasts until the end of skeletal longitudinal growth. Bone remodeling is a life-long process, involving cycles of bone resorption and bone formation; it has a major role in calcium homeostasis, bone adaptation to mechanical stresses and altered bone repair. Bone remodeling is an asynchronous process occurring all over the skeleton to replace micro-damaged bone [14,15]. Schematically, a remodeling cycle follows four steps: 1—activation of a formerly quiescent bone surface and recruitment of osteoclastic precursors; 2—resorption of damaged bone by mature osteoclasts; 3—recruitment of preosteoblasts; 4—new bone formation and mineralization by mature osteoblasts. Bone mass maintenance throughout life is allowed by the strict balance between bone resorption and formation that are linked in time and space. The strict correlation between bone resorption and formation is called coupling, with the formation of an adequate number of osteoblasts at the resorption sites [16].

## 2. Skeletal Evolution throughout Life

Throughout life, bone mass evolves in three phases according to a shifting balance in bone remodeling. During the growth phase, bone formation exceeds bone resorption and osteogenesis predominates. In both males and females, once peak bone mass is reached around the age of 30 years, net bone mass slowly but steadily declines [17]. Because of aging and a decrease in sex hormone secretion, bone resorption becomes higher than bone formation, contributing to osteoporosis and increased fracture risk, more markedly in women after menopause [18]. Estradiol and testosterone evolution curves follow almost the same layout as the bone mineral density evolution curve, which suggests a relationship between both parameters [19].

### 2.1. Skeletal Development

Skeletal mineralization begins during intrauterine life through intramembranous and endochondral ossification and is mainly regulated by the growth hormone/insulin-like growth factor axis [17,20]. Bone mineral mass is acquired relatively slowly throughout childhood, with no substantial sex difference in the axial (lumbar spine) or appendicular (radius and femur) skeleton, when adjusted for age, nutrition and physical activity [21]. Estrogen and androgen regulation is at the basis of the development and maturation of the adult skeleton until epiphyseal fusion, usually by the end of the second decade of life [22]. The peak in bone accretion is reached shortly after peak height gain, at 12.5 ± 0.90 years in girls and 14.1 ± 0.95 years in boys of European ancestry. This asynchrony between the acceleration of standing height and bone mineral content (BMC) or areal bone mineral density (aBMD) is associated with a transiently thinner and less dense cortex with an increased porosity, that may contribute to the male adolescent increased incidence in forearm fractures [21,23].

Peak bone mass (PBM), which is 60 to 80% genetically determined, is achieved by the end of skeletal development during adolescence and early adulthood, depending on skeletal sites and sex [20]. The National Health and Nutrition Examination Survey 2005–2014 showed that attainment of peak BMD occurred significantly earlier in the femoral neck than lumbar spine and earlier in females than males (respectively, between 18.7 and 20.1 years and between 20.5 and 23.6 years) [24]. In spite of the similar volumetric bone mineral density between the two genders during young adulthood, the sexual dimorphism is expressed in bone length, BMD and geometry, providing men with a potential advantage in bone mechanical resistance compared to women [25,26,27]. Men develop wider bones due to greater periosteal expansion and a wider medullary cavity, with higher trabecular volume and cortical porosity than women. However, volumetric BMD is higher in women in the lumbar spine and femoral neck [28,29]. Throughout growth, trabecular bone density increases by way of the thickening of trabeculae and gains in density. These thicker trabeculae may explain the gender and site differences in trabecular volume across adolescence: bone volume per trabecular volume (BV/TV) at the distal radius did not change in girls although it increased in boys [25]. These gender differences are mainly related to the later onset of puberty with a longer duration of prepubertal—predominantly appendicular—growth, and slightly to the greater peak height velocity and the longer duration of the growth spurt in boys compared with girls [28].

**Figure 1:**
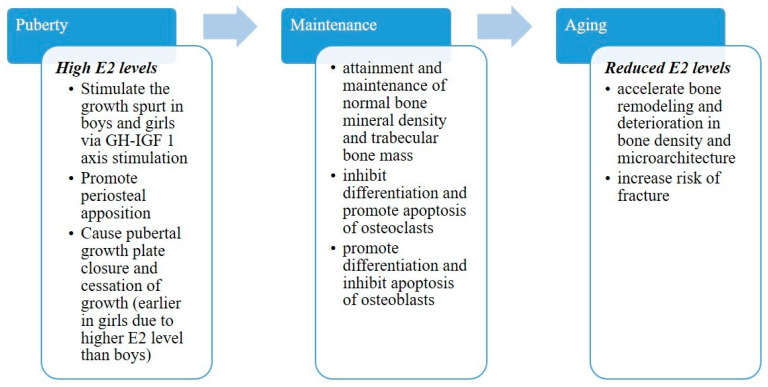
Roles of estrogen in women and men throughout life. (E2: 17β-estradiol).

Estrogens have a biphasic effect on long bone development, which is induced at low concentrations. However, at high doses, estrogens promote epiphysial closure via direct action on proliferating chondrocytes and thereby stop further growth [30] (Figure 1). In humans, the absence of estrogens is associated with unfused growth plates and indeterminate growth [31,32]. Similarly, a young man with an inactivating mutation of estrogen receptor alpha was abnormally tall with unfused epiphyseal growth plates and ongoing growth [33]. Estrogens are also essential for the anabolic action of androgens, as demonstrated in aromatase-deficient men who showed osteopenia and unfused epiphyses [3,34].

Peak bone mass is one of the most important predictors for bone strength and osteoporotic fracture risk later in life. Bone mass acquired at the end of the growth period appears to be more important than bone loss occurring during adult life [35].

### 2.2. Skeletal Maintenance

During the maintenance phase, estrogens and androgens have a slowing effect on osteoclast and osteoblast differentiation and therefore bone remodeling. Bone formation and resorption are in balance. In normal human adults, the remodeling process in a particular site lasts between 6 and 9 months and 10% of the skeleton is replaced every year [36]. Testosterone continues to stimulate periosteal growth, whereas estrogens are important for the maintenance of trabecular bone mass and structure [37]. During the third decade, cortical modeling of long bones with ongoing cortical mineralization, decreasing porosity and endosteal contraction contribute to bone consolidation in men [38,39]. The cortical bone area continues to increase until age 60–70 in men. Conversely, in women, cortical perimeter and bone strength do not increase [38]. Endosteal expansion is greater from age 50 and causes bone loss in older women despite periosteal apposition.

During adulthood, some endocrine pathologies involving estrogens, like complete androgen insensitivity syndrome (CAIS), due to complete androgen resistance, or premature ovarian insufficiency or Turner syndrome, can lead to decreased BMD in women at the lumbar level and at the femoral neck [40,41]. Bone loss associated with estrogen deficiency following menopause is greater in trabecular than in cortical bone [42].

### 2.3. Pregnancy and Lactation

Female bone physiology must adapt to meet the calcium needs of the fetus during pregnancy and then during lactation. During pregnancy, the peak of fetal requirement for minerals occurs in the third trimester. The predominant maternal adaptation to fetal requirement is based on a twofold increase in intestinal calcium absorption. Estrogens increase up to 100-fold during pregnancy but the effects of these hormones on bone homeostasis in pregnant women have been under-explored [43]. Most human data indicate a neutral effect of pregnancy on the maternal skeleton or even greater BMD in long bone [43,44]. Moreover, there is a positive association between the number of pregnancies and fracture risk at the beginning of menopause, as fracture risk reduction becomes statistically significant after three pregnancies [45].

In contrast to pregnancy, the main adaptation during lactation is a temporary demineralization of the skeleton with osteoclast-mediated bone resorption and osteocytic osteolysis. This increased bone turnover is hormonally programmed with reduced estrogen levels and increased Parathyroid Hormone-related protein (PTHrp) levels in breast milk, and is independent of dietary calcium intake. This transient bone loss is reversed after weaning, with a neutral or even protective effect of breastfeeding against low BMD and fragility in long bone, unless exclusive breastfeeding is extended [46,47].

Most studies in pregnant or breastfeeding women explored peripheral bones. However, some studies reported vertebrae osteoporosis and spinal fractures in pregnant women. Even if pregnancy- or lactation-related osteoporosis is rare, it can cause severe back pain, height loss and disability. The prevalence, etiology and physiopathology of this disease are still poorly defined and require further investigation [48,49,50].

### 2.4. Skeletal Involution

BMD along with cortical and cancellous bone mass progressively decline from the fourth decade [51]. Bone loss occurs in response to both estrogen and androgen deficiency and cellular senescence. In women, bone loss is markedly accelerated during the menopause transition, a 3-year period around the final menstrual period, and continues at a lower rate, whereas it is more gradual in men [18,52,53,54]. It may lead to osteoporosis, a skeletal disorder characterized by compromised bone strength predisposing to an increased risk of fracture [51]. In menopausal women, the loss of cancellous bone in the spine accelerates more than in the hip and total body [28]. Men have markedly stronger bones and a lower incidence of osteoporotic fractures because of higher PBM and greater long bone width, along with lower bone loss later in life [28].

### 2.5. Sex Steroid Deficiency

In women, the abrupt decline in serum estradiol levels through menopause is closely associated with an increased osteoclastic bone resorption. Low estrogen levels in postmenopausal women stimulate circulating macrophages to produce osteoclastic cytokines that activate RANK and promote osteoclast activation [17]. Moreover, the loss of the direct pro-apoptotic effects of estrogens on osteoclasts results in the prolongation of osteoclast lifespan, leading to the acceleration of trabecular bone loss [55].

A late menarche or an early menopause has been associated with a significantly higher risk of hip fracture. After adjustment for other variables, a relatively earlier menarche was associated with a slightly better protective effect than a relatively later menopause, whereas late puberty was associated with non-maximum bone acquisition and higher fracture risk [21,56,57,58]. The Women’s Health Initiative Observational Study concluded that higher bioavailable estradiol and testosterone in postmenopausal women were associated with a decreased risk of osteoporotic fracture [59]. Conversely, bone loss in men is more due to decreased osteogenesis, which results in thinner, less perforated trabeculae, which are more widely spaced compared to those of postmenopausal women. Estrogens are key regulators of bone metabolism not only in women but also in men. Their declined serum level with age in men are correlated with decreasing BMD levels [60,61] (Figure 1). Interestingly, gender-affirming hormone therapy (GAHT) in trans women (with estradiol and antiandrogens) and in trans men (with testosterone) resulted in increased bone turnover in younger trans men whereas it decreased in trans women and older trans men (postmenopausal), showing the crucial role of estrogen in bone resorption regulation [62]. In men, fractures have been shown to be inversely associated with bioavailable free testosterone (but not with total testosterone) when the sex hormone-binding globulin (SHBG) level was high [63].

### 2.6. Aging

Bone resorption results from increased osteocyte apoptosis, a reduced number of osteoblast precursors and stem cells from which these precursors are derived or reduced osteoblast lifespan [19,36]. Additionally, increased glucocorticoid production and sensitivity with advancing age decrease skeletal hydration and thereby increase skeletal fragility [36].

With aging, cortical porosity increases more in women than in men, while trabecular thickness declines more in men than in women, and bone expansion at the periosteal surface increases more in men than in women so that the total bone area becomes larger in men than in women [64]. Older women show dramatically greater medullary expansion and cortical thinning, due to endocortical resorption outpacing periosteal apposition [65].

## 3. Roles of Estrogen Receptors

### 3.1. Estrogen Receptors

Estrogen effects are mediated by two receptors, estrogen receptors alpha (ERα) and beta (ERβ). Encoded by gene *esr1* and cloned in 1986, ERα has been considered as the unique receptor for estrogens until the discovery and cloning of ERβ, encoded by gene *esr2*, ten years later [66,67,68,69]. Although structurally closely related, expression patterns of estrogen receptors are different since ERα is widely expressed not only in reproductive organs in females and males (uterus, mammary gland, testes, epididymis) but also in non-reproductive organs such as the liver, heart and muscles; ERβ is mainly expressed in ovaries and the prostate gland. However, both receptors are expressed in bone tissue [2,70]. As members of the nuclear receptor superfamily, ERα and ERβ present six structural domains, A to F, a DNA-binding domain (DBD) and a ligand binding domain (LBD), as well as two Activating Functions, AF1 and AF2, involved in cofactor recruitment and in fine chromatin remodeling and gene transcription [71].

**Figure 2:**
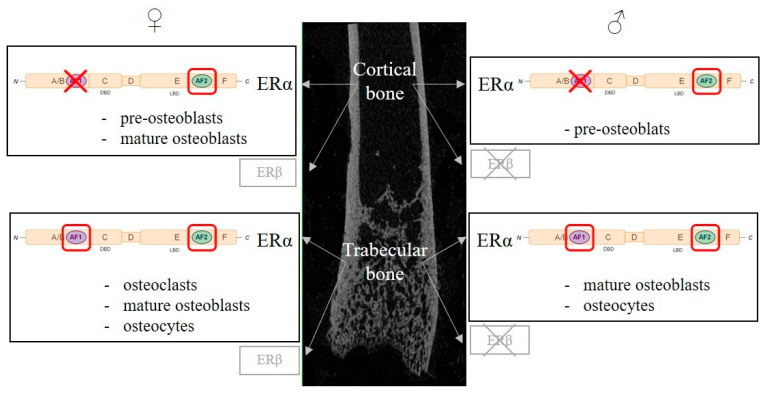
Regulation of bone metabolism by estrogen receptors, cellular and molecular aspects. Estrogen’s protective effects on trabecular and cortical bone are mainly mediated by Estrogen Receptor α (ERα) in both females and males, while ERβ only plays a minor role in female and none in male. ERα belongs to the nuclear receptor superfamily and exerts its transcriptional activity though two activating functions (AFs), AF1 and AF2. Both AF1 and AF2 functions are necessary to mediate estrogen effects, whereas, in the cortical compartment, only AF2 function is necessary in females and males. Genetic murine models have allowed the study of the role of ERα in bone cells (osteoclasts, osteoblasts and osteocytes). For each bone compartment and sex, the cell types involved in estrogen’s protective effects are indicated. A red box highlights essential ERα subfunctions in each cell type, whereas a red X indicates the dispensable ERα subfunction.

Murine models, deficient for ERα and ERβ, have allowed the study of the respective roles of each receptor in bone tissue. Results from the first studies, published in the early 2000s, have to be considered with caution since the murine model initially used (ERα and ERβ-*Neo*-KO) are not complete functional knockouts and still express truncated forms of ERα able to bind E2 [72,73]. New models, totally deficient in ERα (ERα^−/−^) and ERβ (ERβ^−/−^), have since been developed [74]. A first study in intact female and male mice showed that ERα deletion was associated with reduced bone turnover and increased trabecular bone volume in both genders. ERβ deletion had the same effects but only in female mice and the deletion of both receptors caused a drop in trabecular bone volume and turnover. ERα^−/−^ mice exhibited strongly increased levels of circulating E2 and testosterone in females and males, resulting from a profound disruption of endocrine feedback loops [75]. To better understand the effects of ER deletion, gonadectomy was performed and associated with a reduction in all bone parameters (BMD, BV/TV, cortical thickness) and an increased bone turnover. E2 systemic administration could not restore bone features in ERα^−/−^ female and male mice in contrast to wildtype (WT). In contrast, ERβ^−/−^ mice had a similar response to E2 as WT [76], showing the prominent role of ERα in bone responses to estrogen in both males and females, while ERβ only has a minor protective role in females and none in males [75,76] (Figure 2). As in long bones and vertebrae, ERα is also necessary for E2’s protective effects in the mandible at alveolar, cortical and trabecular sites, whereas ERβ is dispensable [77]. ERαβ^−/−^ mice, allowing the study of the possible compensatory role of one ER or the other in single-gene KO models (ERα^−/−^ and ERβ^−/−^), exhibit a similar bone phenotype to ERα^−/−^ mice, showing that ERβ is not sufficient to compensate for ERα actions in ERα^−/−^ mice and that ERα is truly the central actor in estrogen osteoprotective effects [76].

However, constitutive ERα inactivation could lead to important developmental changes that may alter the adult mouse phenotype, although ERα appears to play a minor, if any, role in the development of most tissues, including those of the reproductive organs. To avoid this bias and study the E2 actions on ERα in adults, a team recently used an ERα-deficient tamoxifen-inducible mouse model [78]. In this model, trabecular responses to E2 treatment after ovariectomy and bone turnover are reduced but cortical response is maintained. Normal cortical responses to E2 in mutant mice may be explained by ERβ involvement either locally in bone tissue or by indirect effects in other tissues. For instance, estrogen receptors are expressed in several brain regions, and E2 is able to regulate cortical bone mass in female mice via indirect central nervous system mechanisms [79,80].

### 3.2. Estrogen Receptors in Bone Cells

The development of mouse models with conditional targeted deletion of ERs using the Cre-loxP system allowed the definition of more precise estrogen cellular targets in bone physiology. The expression of a Cre-recombinase controlled by the promotors of genes expressed specifically in targeted cell types or tissues results in the deletion of a genomic region of interest (here, crucial sequences of *Esr1* or *Esr2*) flanked by two LoxP sites. Several models have been used to mainly study the role of ERα but also that of ERβ in bone cells (Table 1, Figure 2).

The deletion of ERα under the control of the cathepsine K (CtsK) promoter, specifically expressed in mature osteoclasts, was associated with trabecular bone loss in 12-week-old female mice but not in males [86]. Ovariectomy caused minor supplemental bone loss and E2 treatment restored trabecular bone mass but had no impact on the cortical compartment. Trabecular bone mass reduction was associated in CtsK-Cre^+^Erα^lox/lox^ mice with increased osteoclast number and bone turnover in trabecular bone. A second model targeting myeloid osteoclast precursors under the control of the lysozyme M (LysM) promoter showed similar results. While female mutated mice exhibited an increased number of osteoclastic progenitors in bone marrow and differentiated osteoclasts in vertebrae, they had altered trabecular bone mass and microarchitecture, but no effect was observed in cortical bone [55]. In contrast, LysM-Cre^+^ERα^lox/lox^ males had a similar bone phenotype to WT mice in both trabecular and cortical compartments [85].

ERα deletion in mesenchymal pluripotent osteoblast progenitors under the control of the *Prx1* gene promoter (thus in all the following differentiation stages) was characterized by reduced cortical bone thickness and periosteal bone formation in female mice [87]. In contrast to WT mice, ovariectomy in Prx1-Cre^+^ERα^lox/lox^ female mice did not induce supplemental cortical bone loss and was not associated with increased osteoclast number at the endocortical bone surface in femurs. In male mice, cortical bone mass was transitorily reduced at 8 weeks of age but was similar to WT at 22 weeks of age. Trabecular bone was unaffected in mutant females and males. Bone marrow cellular culture from Prx1-Cre^+^ERα^lox/lox^ mice showed reduced osteoblast number and activity as evidenced by reduced expression of osteoblast differentiation markers *Runx2*, *Osx*, *Col1a1* and *Bglap* [87]. ERβ deletion at the same stage of osteoblastic differentiation was associated with increased trabecular bone mass in female mice [89]. Female mice with ERα inactivation at a more advanced differentiation stage, in osteoblast progenitors present in bone-forming regions, under the control of the *Osx1* (also called *Sp7*) gene promotor, had a similar bone phenotype and osteogenesis alteration [87]. Male Osx1-Cre^+^ERα^lox/lox^ mice also had transiently reduced cortical bone mass and trabecular bone was not affected [85]. Transiently reduced cortical bone mass in males seems to reflect a delay in cortical bone mass acquisition during puberty since adult mice have normal cortical bone mass, suggesting that the action of androgens mediated by androgen receptors compensates for the absence of ERα. Finally, the targeted deletion of ERα in terminal osteoblastic differentiation in mature osteoblasts and osteocytes, controlled by the *Col1a1* gene promoter, had no effect on trabecular and cortical bone in neither female nor male mice [87]. However, other teams invalidated ERα in mature osteoblasts using a different gene promoter encoding for OCN, and had different results [81,82,88]. In these studies, mutant female mice exhibited reduced trabecular bone mass and cortical thickness in femurs, tibia and vertebrae and reduced bone turnover marked by a limited number of osteoblasts and osteoclasts. No effect of ERα inactivation was observed in male mice [81,82,88]. These discrepancies may be explained by the use of different Cre models in which promoters are engaged in different chronological expression cellular effects. The Col1a1-Cre model targets osteoblasts during bone matrix maturation and Ocn-Cre targets osteoblasts later on, during bone matrix mineralization. Finally, ERα inactivation in osteocytes using Dmp1-Cre was associated with conflicting results [83,84]. One study found reduced trabecular bone mass and no cortical alteration [84], whereas another showed no bone phenotype in intact 12-week-old female mice [83]. This difference may be explained by the use of ERα-floxed mice with different genetic backgrounds. Moreover, it is important to stress that a recent study using flow cytometry and high-resolution microscopy showed that Ocn-Cre and Dmp1-Cre models do not only target mature osteoblasts and osteocytes but also affect broader stromal cell populations than initially considered. Results from Ocn-Cre+ERα^lox/lox^ and Dmp1-Cre^+^ERα^lox/lox^ mice must then be interpretated with caution [90].

An alternative approach to study the role of ERα in bone tissue consists in the use of hematopoietic chimeras. The reconstruction of lethally irradiated ERα^−/−^ mice with bone marrow from ERα^+/+^ mice shows that ERα is necessary in non-hematopoietic cells, including osteoblasts, to mediate E2 effects on trabecular and cortical bone compartments [91]. A reverse bone marrow transplant from ERα^−/−^ into WT mice showed that E2 effects on cortical and trabecular bone are enhanced by ERα in the hematopoietic compartment, suggesting that ERα expression in hematopoietic cells potentiates E2 bone-protective effects but only in the presence of ERα in non-hematopoietic cells [91]. Conflicting results with cell-specific inactivation of ERα using the Cre-Lox system may be explained by the fact that bone marrow transplants involve other cells than osteoblasts and osteoclast precursors. Indeed, several cell types of hematopoietic origin, beside osteoclasts, are involved in the estrogenic regulation of bone mass, including T and B lymphocytes [92,93]. A recent study showed that E2 effects on T lymphocytes are indirect since specific ERα deletion under the control of the *Lck* gene promoter had no effect on trabecular and cortical bone responses to ovariectomy and E2 treatment in female mice [94]. Moreover, mice with a deletion of ERα specifically in B lymphocytes have a similar bone phenotype to normal controls [95]. Results from the last two studies show that ERα signaling in T and B cells seems to be dispensable for bone loss caused by estrogen deficiency and that E2 effects on bone do not directly target those cells types and are more likely to be indirect.

The use of conditional deletion mice models of ERα in different bone cells showed that estrogen’s protective effects on trabecular and cortical bone compartments involve different cell types. E2 bone-protective effects on trabecular bone are mediated via direct actions on osteoclasts in female mice. ERα in osteoblast progenitors has a major role in cortical bone mass acquisition in female mice, whereas ERα in osteoblast mesenchymal precursors is involved in estrogen’s protective actions against endocortical bone resorption.

### 3.3. Nuclear vs. Non-Nuclear Erα-Mediated Pathways

In its inactive state, ERα is distributed in the nucleus, cytoplasm and plasma membrane in varying proportions, depending cell type. Ligand fixation on the receptor induces two major signaling pathways, nuclear/genomic ERα and membrane/non-genomic ERα.

In nuclear-initiated pathways, ligand fixation induces ERα dimerization and translocation to the nucleus, but in numerous cells, significant amounts of ERα appear to be present in the nucleus even in the absence of any ligand. In the nucleus, the ERα dimer interacts with specific promotors of target genes on precise sequences called estrogen response elements (EREs; ERE-dependent, “classical” pathway) or in interaction with other transcription factors such as AP-1 or SP1 bound to very specific DNA sequences (ERE-independent pathway). A third nuclear-initiated pathway is ligand independent, as ERα can be indirectly activated by growth factors like EGF or IGF-1. The fixation to their respective transmembrane receptor activates intracellular kinases able to phosphorylate ERα, modulating its interactions with specific cofactors [71]. ERα AF1 and AF2’s transactivating functions are able to act either synergically or independently to control target gene transcription. AF1 and AF2’s activities are finely controlled by transcription cofactor availability, cell type and the nature of the regulated promotor [71]. In order to study the respective roles of AF1 and AF2, mice models lacking one or the other have been developed [96,97]. In bone tissue, ERαAF1 is necessary to mediate E2’s protective effects on trabecular bone in both females and males but is only partly necessary in the cortical compartment. ERαAF2 is necessary in both the cortical and trabecular compartment to elicit full E2 effects in vertebrae and femurs in both genders; in the mandible, E2’s effects on alveolar, trabecular and cortical compartments are also mediated by ERαAF2 when a dose effect can be studied precisely thanks to an appropriate pellet to deliver accurate E2 doses [77,98,99,100] (Table 2, Figure 2).

Besides classically described nuclear effects, ERα signaling pathways can be initiated at the plasma membrane (membrane-initiated steroid signaling, MISS). Membrane signaling of steroid receptors has been shown in several cell types, including osteoblasts and osteoclasts [104]. Two murine models have been developed to investigate the physiological roles of ERα-MISS in vivo. The first one consists in a point mutation of ERα at its palmitoylation site, necessary for its addressing to the plasma membrane, and C451A-ERα mice exhibit a membrane-specific loss of function of ERα [105,106]. Two studies show that in the axial skeleton, E2’s effects on trabecular bone are strongly dependent on membrane ERα (mERα) whereas in long bones, cortical and trabecular E2’s effects are only partly dependent on mERα in growing and adult female mice [102,103]. Moreover, ERα-MISS seems to impact osteoblasts but not the osteoclast lineage in response to E2 [102]. In the mandible, bone response to E2 treatment is slightly but significantly reduced in alveolar, trabecular and cortical compartments [77]. These results show that mERα is necessary to elicit full E2 bone-protective effects. The second model, R264A-ERα, consisting in a point mutation of ERα in a sequence necessary for its interaction with proteins at the plasma membrane, is fertile in contrast to C451A-ERα but unable to display classical membrane-initiated E2 effects (accelerated reendothelialization, arterial dilation) [107]. Surprisingly, R264A-ERα mice exhibit similar bone responses to ovariectomy and E2 treatment to WT (BMD, cortical thickness, trabecular bone mass) [108]. Thus, it seems that the functional consequences of this second ERα point mutation appear to be restricted to endothelial cells, without impacting on the mERα in other cell types.

Besides the use of genetically modified models, another approach to study the respective roles of nuclear and membrane ERα is pharmacological. Despite a weak agonist activity for ERα and ERβ, estetrol (E4), an estrogen produced by fetal liver, has similar effects to E2 on uterine gene expression and epithelial proliferation when administered in high doses to female mice and prevents atheroma. All these E4 actions are known to involve nuclear ERα action, whereas E4 is unable to activate endothelial (NOS) and to accelerate endothelial healing, which are MISS-dependent effects [109,110,111,112]. Thus, E4 is classified as a natural estrogen with selective action in tissues (NEST) displaying nuclear ERα activation only. E4 administration to osteoporotic female rats was associated to increased bone mineral density and bone strength in a dose-dependent manner [113]. E4 has been studied in human trials as a candidate for menopause hormonal therapy; besides estrogenic effects on reproductive tissues (vaginal epithelium, endometrium) and hot flushes, it has dose-dependent estrogenic effects on bone with a reduction of osteocalcin (bone formation marker) and CTX-1 (bone resorption marker) serum concentrations in postmenopausal women [1,114,115]. As part of studies regarding prostate cancer, E4 administration to healthy men reduces bone turnover markers, although not significantly for osteocalcin [116].

While E4 activates ERα nuclear pathways, two other chemical compounds are able to only elicit ERαMISS, estrogen–dendrimer conjugate (EDC) and pathway preferential estrogens (PaPEs). EDC consists of ethinyl-estradiol attached to a large, positively charged, nondegradable poly(amido)amine dendrimer preventing it from translocating to the nucleus [117]. EDC promotes endothelial protection in mice since it increases NO production and accelerates reendothelization without inducing uterine or breast cancer growth [118]. In bone tissue, EDC administration is associated with increased femoral and vertebral cortical bone mass and bone strength. However, it does not seem to have effects on vertebral trabecular bone in either female or male mice [101,119]. In the mandible, EDC increases alveolar bone mass but has no effects on trabecular and cortical bone [77]. More recently, PaPEs originating from the rearrangement of E2 steroidal structure have been developed. PaPEs form complexes with ER with a very short lifespan, sufficient to selectively activate membrane-initiated ER pathways but too transient to maintain nuclear activity; they exert beneficial effects in metabolic tissues and the vasculature [120]. PaPEs have similar effects on mandibular bone to EDC, with increased alveolar bone mass and no effects on trabecular and cortical bone in female mice [77]. PaPEs’ effects on vertebral and femoral bone in both females and males have not been determined yet.

Results from genetical approaches targeting nuclear loss-of-function ERα-AF2° on the one hand, and pharmacological approaches with EDC and PaPEs on the other hand, may seem contradictory. Selective activation of ERαMISS with EDC has effects on long and mandibular bone [77,101] while E2’s beneficial actions are totally abrogated in nuclear ERα-AF2°-deficient mice [99]. Nevertheless, it has been shown that nuclear ERα-AF1 is necessary for EDC effects, emphasizing the importance of the crosstalk between nuclear and membrane ERs to relay estrogen’s beneficial effects on bone [121]. Moreover, one can imagine that part of EDC and PaPEs’ actions could involve an activation of both AF1 and AF2, implying another level of interaction between nuclear and membrane ERα.

### 3.4. Selective Estrogen Receptor Modulators

Depending on the ligand nature, ERs adopt a specific conformation. Following the fixation of an agonistic ligand such as E2, the position of helix 12 determines the formation of an AF2 region available for transcriptional coactivators to bind on to. In contrast, when an antagonist binds to the receptor, the helix 12 position blocks cofactor recruitment and prevents gene transcription. Between those extremes, the receptor–ligand complex adopts a unique conformation for each ER ligand [71]. Selective estrogen receptor modulators (SERMs) are synthetic pharmacological compounds, lacking an estrogen steroidal structure but exhibiting a tertiary structure, able to bind onto ERs; they can selectively elicit estrogen’s protective effects (on bone tissue, the cardiovascular system or metabolism) without triggering deleterious impacts (after menopause, on uterus or mammary glands). Several SERMs are thus employed in clinical practice to treat and prevent breast cancer, osteoporosis or menopause symptoms [122,123]. Tamoxifen (Tam), was developed in the 1970s for breast cancer treatment. It increases cortical and trabecular bone mass in both female and male intact mice and partially blocks orchidectomy-induced trabecular bone loss in male mice [124,125,126,127]. Raloxifene (Ral) is approved for the prevention and treatment of osteoporosis in postmenopausal women since it reduces the risk of vertebral fractures by 30–50% [128]. Its administration increases trabecular bone mass and mineral density in male mice and enhances vertebral trabecular bone mass and femoral cortical bone mass in female mice [98,127,129]. More recently, two other SERMs, lasofoxifen (Las) and bazedoxifene (Bza) have been approved in Europe for the treatment or postmenopausal osteoporosis; they decrease the risk of vertebral as well as non-vertebral fractures [130,131]. In mice, whereas Las increases trabecular bone mass in the axial skeleton and both trabecular and cortical bone mass in the appendicular skeleton, Bza only enhances trabecular bone mass in the axial skeleton in both genders [98,127,129]. It has been recently shown that ERα-AF1 is necessary for the bone estrogenic effects of Ral, Las and Bza in female and male mice [98,129].

### 3.5. Mechanical Loading

Mechanical strains are considered to be a critical regulator of bone homeostasis; they determine bone shape, structure and mass and affect bone remodeling in favor of bone formation. For example, mechanical unloading resulting from weightlessness during space flights is associated with reduced trabecular and cortical bone mineral density and accelerated bone resorption [132]. Thus, during growth, children with moderate physical activity exhibit greater bone mineral content than sedentary children [133].

Regarding the osteogenic effects of mechanical loading, several studies reveal an implication of ERα that would act independently of any ligand. Indeed, the application of mechanical tensions on osteoblast and osteocyte cultures promotes their activation and proliferation via ERα [134,135]. Moreover, a study about the effects of the interactions between ERα gene polymorphism and physical activity on bone mass modulation in humans suggests that genetic variants of the *esr 1* locus could modulate bone tissue mechanosensitivity, supporting a major role of bone cell ERα in bone adaptation to mechanical strains [136].

Several studies showed that ERα is involved in vivo in the osteogenic effects of mechanical strains in a ligand-independent manner [137,138]. In female ERα^−/−^ mice, osteogenic response to mechanical loading is reduced in cortical but not trabecular bone compared to WT; conversely, in males, ERα inactivation is associated with increased osteogenic response to mechanical loading in both bone compartments. ERβ^−/−^ female and male mice exhibit increased osteogenic responses to loading in cortical but not trabecular bone [137]. In that respect, ERα and ERβ seem to have opposite effects and may be in competition for the regulation of bone remodeling by mechanical strains in female mice. Finally, ERα-AF1 is necessary to mediate osteogenic effects of mechanical loading, whereas ERα-AF2 is not required in female mice [138]. This again suggests a key role of E2-independent actions of ERα, potentially through activation of ERα-AF1 that is the target of growth factors. The involvement of ERα in the mechanical strains of bone homeostasis should undoubtedly be further studied in the future to optimize the approaches to fight bone demineralization and the risk of bone fracture.

## 4. Conclusions

In the last ten years, tremendous advances have been made in the knowledge of estrogens and estrogen receptor functions in bone but also in other tissues. The progress in genetic mouse models and pharmacological engineering has allowed us to begin to decipher mechanisms of actions of ERs and should pave the way to optimize selective ER modulation.

## Figures and Tables

**Table 1:** ERα role in bone cells and the impact of its selective deletion in trabecular and cortical bone compartments in female and male mice.

Targeted Cells	Osteoclasts				Osteoblasts								Osteocytes	
Differentiation Stage	Myeloid Progenitors		Mature Osteoclasts		Pluripotent Mesenchymal Progenitors		Osteoblastic Progenitors		Mature Matrix Maturation		Mature Mineralization		NA	
**gene promotor**	***LysM***		***CtsK***		***Prx1***		***Osx1***		***Col1a1***		***Ocn***		***Dmp1***	
**gender**	female	male	female	male	female	male	female	male	female	male	female	male	female	male
**trabecular bone**	↘	↔	↘	↔	↔	↔	↔	↔	↔	↔	↘	↔ [81] ↘ [82]	↔ [83] ↘ [84]	↘
**cortical bone**	↔	↔	↔	↔	↘	↘ *	↘	↘ *	↔	↔	↘	↔	↔	↔
**references**	[55]	[85]	[86]	[86]	[87]	[87]	[87]	[85]	[87]	[87]	[81,82,88]	[81,82]	[83,84]	[83]

↔ no effect; ↘ bone mass reduction; * transient effect.

**Table 2:** Genetic and pharmacological approaches to study the roles of ERs and their subfunctions in bone tissue regulation.

		Trabecular Bone		Cortical Bone		Alveolar Bone		
Mouse Model	Treatment	Female	Male	Female	Male	Female	Male	References
WT	E2	↗↗	↗↗	↗↗	↗↗	↗↗	NT	[76,77]
	EDC	↔	NT	↗	NT	↗	NT	[77,101]
	PaPEs	NT	NT	NT	NT	↗	NT	[77]
ERα^−/−^	E2	↔	↔	↔	↔	↔	NT	[76,77]
ERβ^−/−^	E2	↗	↗↗	↗	↗↗	↗↗	NT	[76,77]
ERα AF1°	E2	↔	↔	↗↗	↗↗	NT	NT	[98,99]
ERα AF2°	E2	↔	↔	↔	↔	↔	NT	[77,98,99]
C451A-ERα	E2	↗	↗	↗	↗	↗	NT	[77,102,103]

↔ no effect; ↗↗ steep bone mass increase; ↗ small bone mass increase; WT: Wild Type; ER: Estrogen Receptor; AF: Activcating Function; E2: 17β-estradiol; EDC: estrogen–dendrimer conjugate; PaPEs: pathway preferential estrogens; NT: not tested.
